# Hybrid argon plasma coagulation for chronic radiation-induced proctitis following pelvic chemoradiotherapy for cervical adenocarcinoma: a case report

**DOI:** 10.1055/a-2686-7678

**Published:** 2025-09-04

**Authors:** Aiji Hattori, Yohei Ikenoyama, Yasuko Fujiwara, Misaki Nakamura, Yasuhiko Hamada, Noriyuki Horiki, Hayato Nakagawa

**Affiliations:** 1 220937 Department of Gastroenterology and Hepatology, Mie University Hospital, Tsu, Japan

Chronic radiation-induced proctitis is a common complication of pelvic radiotherapy that frequently presents with rectal bleeding and mucosal friability. Argon plasma coagulation (APC) is considered the first-line treatment for this condition 1 ; however, it is associated with complications, such as rectal pain, ulceration, and perforation 2 3 . Hybrid APC (hAPC), which combines high-pressure submucosal fluid injection with thermal ablation 4 , has shown promise in achieving hemostasis while minimizing the depth of thermal injury to the muscular layer ( Fig. 1 ). Here, we report a case of chronic radiation-induced proctitis successfully treated with hAPC.

**Fig. 1:**
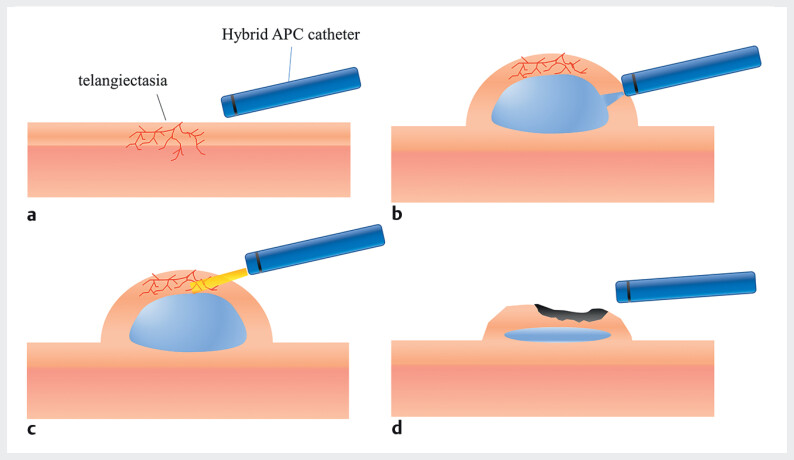
The schema of hybrid argon plasma coagulation (hAPC). **a** The APC catheter was attached to the mucosa. **b** Submucosal fluid cushion was created by high-pressure injection without a needle. **c** After injection, thermal ablation was carried out to the telangiectasia. **d** The presence of submucosal cushions helped ensure the safety of the thermal ablation procedure.

A woman in her 50s with a history of laparoscopic radical hysterectomy and chemoradiotherapy for cervical adenocarcinoma presented two years later with sintermittent hematochezia. Colonoscopy revealed diffuse vascular ectasia with an oozing hemorrhage in the rectum ( Fig. 2 ). The patient was diagnosed with chronic radiation-induced proctitis and hAPC was performed ( Video 1 ).

**Fig. 2:**
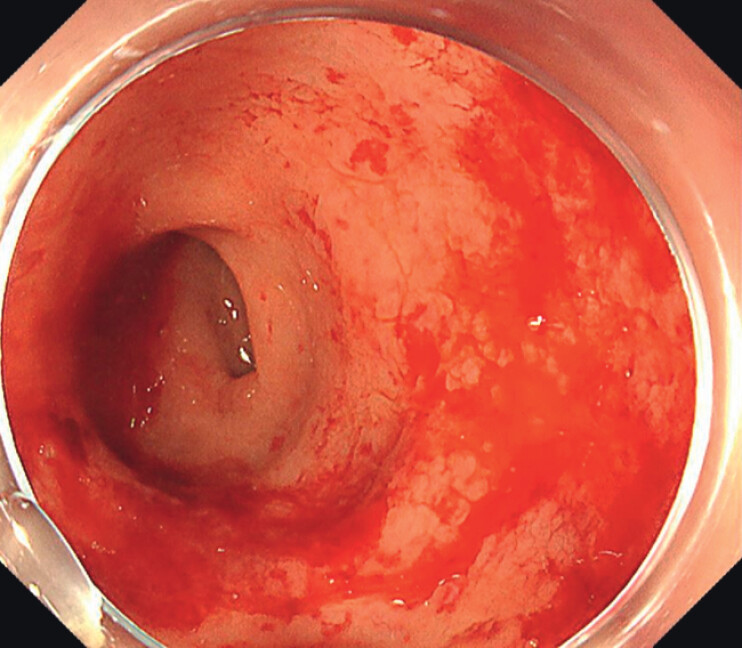
The chronic radiation-induced proctitis in the rectum before hAPC treatment. There was diffuse vascular ectasia with an oozing hemorrhage in the rectum.

Hybrid argon plasma coagulation procedure for chronic radiation-induced proctitis.Video 1

Treatment was performed using an APC catheter connected to an electrosurgical unit (VIO APC 3; Erbe, Tübingen, Germany) and a needleless high-pressure waterjet system (ERBEJET 2). First, a submucosal fluid cushion was created by injecting saline containing indigo carmine. The tip of the catheter was slightly pressed against the mucosa to ensure stability and the solution was injected. After injection, thermal ablation was performed in the affected areas (using the forced APC effect 4). The presence of a submucosal cushion ensured safe thermal ablation. We ablated all visible telangiectasia. The patient reported no pain or adverse effects following the procedure. Follow-up colonoscopy performed seven days after treatment revealed no visible bleeding ( Fig. 3 ).

**Fig. 3:**
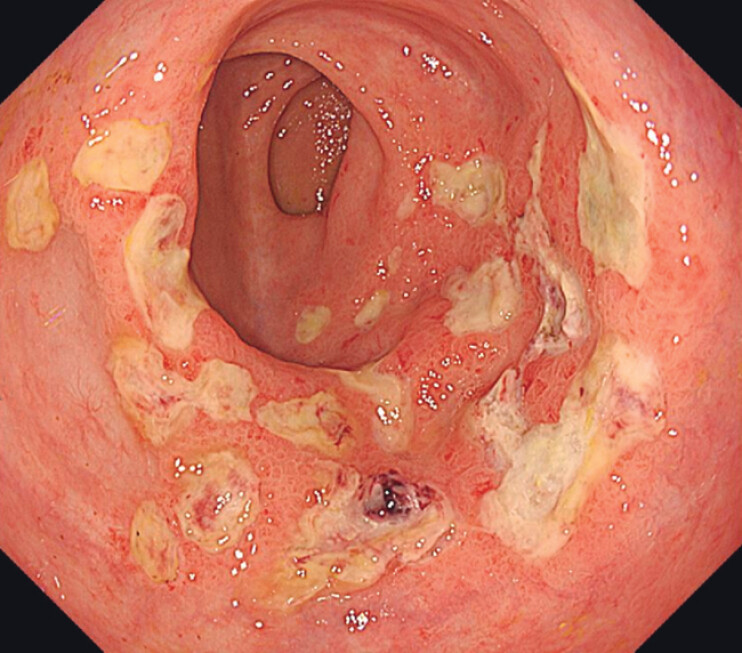
The rectal lesion after hAPC treatment. Follow-up colonoscopy performed seven days after hAPC revealed diffuse post-treatment erosions but no visible bleeding.

To our knowledge, this is the first report of the use of hAPC for radiation proctitis. Thus, hAPC may be a highly effective and safe treatment option, particularly for bleeding caused by telangiectasia, as observed in the present case.

Endoscopy_UCTN_Code_TTT_1AQ_2AZ

## Notes

Endoscopy E-Videos https://eref.thieme.de/e-videos
*E-Videos* is an open access online section of the journal *Endoscopy* , reporting on interesting cases and new techniques in gastroenterological endoscopy. All papers include a high-quality video and are published with a Creative Commons CC-BY license. Endoscopy E-Videos qualify for HINARI discounts and waivers and eligibility is automatically checked during the submission process. We grant 100% waivers to articles whose corresponding authors are based in Group A countries and 50% waivers to those who are based in Group B countries as classified by Research4Life (see: https://www.research4life.org/access/eligibility/ ). This section has its own submission website at https://mc.manuscriptcentral.com/e-videos .
